# Progesterone Increases Apoptosis and Inversely Decreases Autophagy in Human Hepatoma HA22T/VGH Cells Treated with Epirubicin

**DOI:** 10.1155/2014/567148

**Published:** 2014-05-19

**Authors:** Wen-Tsan Chang, Hsiao-Ling Cheng, Bau-Shan Hsieh, Chien-Chih Chiu, King-Teh Lee, Kee-Lung Chang

**Affiliations:** ^1^Graduate Institute of Medicine, College of Medicine, Kaohsiung Medical University, Kaohsiung 80708, Taiwan; ^2^Division of Hepatobiliary and Pancreatic Surgery, Department of Surgery, Kaohsiung Medical University Hospital, Kaohsiung 80756, Taiwan; ^3^Department of Surgery, School of Medicine, College of Medicine, Kaohsiung Medical University, Kaohsiung 80708, Taiwan; ^4^Department of Biochemistry, School of Medicine, College of Medicine, Kaohsiung Medical University, Kaohsiung 80708, Taiwan; ^5^Department of Biotechnology, College of Life Science, Kaohsiung Medical University, Kaohsiung 80708, Taiwan

## Abstract

Hepatocellular carcinoma (HCC) is the leading cause of cancer-related deaths worldwide. Epirubicin can induce intracellular reactive oxygen species and is widely used to treat unresectable HCC. Progesterone has been found to inhibit the proliferation of hepatoma cells. This study was designed to test the combined effects of epirubicin and progesterone on human hepatoma cell line, HA22T/VGH. These cells were treated with different concentrations of epirubicin with or without the coaddition of 30 **μ**M progesterone and then analyzed for apoptosis, autophagy, and expressions of apoptotic-related proteins and multidrug-resistant gene. Epirubicin treatment dose-dependently inhibited the growth of HA22T/VGH cells. Addition of 30 **μ**M progesterone, which was inactive alone, augmented the effect of epirubicin on the inhibition of growth of HA22T/VGH cells. Cotreatment with progesterone enhanced epirubicin-induced apoptosis, as evidenced by greater increase in caspase-3 activity and in the ratio of the apoptosis-regulating protein, Bax/Bcl-X_L_. The combination also caused a decrease in autophagy and in the expression of multidrug resistance-related protein 1 mRNA compared to epirubicin alone. This study shows the epirubicin/progesterone combination was more effective in increasing apoptosis and inversely decreasing autophagy on HA22T/VGH cells treated with epirubicin alone, suggesting that this combination can potentially be used to treat HCC.

## 1. Introduction

Hepatocellular carcinoma (HCC) is the fifth most common cancer in men and the seventh in women, and it is the third most common cause of cancer-related deaths worldwide [1, 2]. Liver resection, local ablation therapy, and liver transplantation are the suggested curative therapies for HCC, while transarterial chemoembolization (TACE) has been used to treat unresectable HCC with some clinical efficacy [3–5].

Anthracyclines, such as doxorubicin or epirubicin, have been widely used to treat advanced HCC, to prevent or treat postoperative recurrence, and to downstage the disease before liver transplantation by systemic infusion or by transarterial route [6, 7]. Acting as topoisomerase-II inhibitors, anthracycline drugs induce DNA damages and acute oxidative stress in cells [8, 9]. And the quinine group of anthracyclines can cause one electron reduction to produce a semiquinone [9, 10]. The free radical semiquinone consequently produces reactive oxygen species (ROS), including superoxide anions, hydrogen peroxide, and hydroxyl radicals [9, 10]. ROS are reported to be involved in epirubicin-induced apoptosis in hepatoma cell lines [10]. Furthermore, epirubicin is less cardiotoxic than doxorubicin [6]. Therefore, epirubicin is widely used in Europe and Asia in the treatments of cancers, including HCC [6, 8].

However, the side effects of anthracyclines include cardiomyopathy, immunosuppression, and the development of primary or secondary drug resistance, which may sometimes adversely affect survival, recurrence, and extrahepatic metastases in HCC patients [11–13]. And HCC cells themselves are usually resistant to chemotherapeutic agents, the response rates of chemotherapy in HCC are reported to be only 10.5%–20.6%, and, generally, overall survivals have been less than 12 months for advanced HCC patients [6, 12].

Autophagy, a survival mechanism of some cancer cells, can be induced during starvation, chemotherapy, radiation, hypoxia, and some endocrine therapies [14, 15]. Sun et al reported that autophagy could protect breast cancer cells from epirubicin-induced apoptosis and facilitate the development of epirubicin resistance [16]. Progesterone can reverse multidrug resistance gene expression in epirubicin-treated urethral cancer cell lines via p-glycoprotein pathway [17]. And megestrol (a progestin drug) treatment has produced some efficacy in advanced HCC in some clinical studies [18, 19]. Therefore, combining epirubicin with progesterone might be a potential strategy for treating HCC, as it might allow for the smaller doses of epirubicin, which is generally toxic, while increasing its effectiveness against HCC and decreasing epirubicin-related side effects.

Our previous study showed HA22T/VGH cells are susceptible to changes in redox status and oxidative stresses also induce apoptosis in these cells [20]. This study tested the effect of combining epirubicin with progesterone to treat a metastatic, poorly differentiated HCC cell line, HA22T/VGH. To evaluate the therapeutic effect of this combination, we analyzed occurrence of apoptosis and autophagy, expressions of their related proteins, and the expression of multidrug resistance-related protein 1 (MRP-1) gene.

## 2. Materials and Methods

### 2.1. Reagents and Antibodies

Epirubicin hydrochloride (Pharmorubicin) was purchased from Pfizer Italia S.R.L. (Milano, Italy), progesterone from Sigma-Aldrich (St. Louis, MO, USA), acridine orange from Molecular Probes (Eugene, OR, USA), protein assay reagents from Bio-Rad Laboratories (Hercules, CA, USA), and TRIzol reagent from Invitrogen Life Technologies (Carlsbad, CA, USA). All other chemicals were of analytical grade and purchased from Sigma-Aldrich (St. Louis, MO, USA). Mouse monoclonal antibodies against light chain-3 (LC-3), Beclin-1, or Bax, rabbit polyclonal antibodies against Bcl-X_L_, and goat polyclonal antibodies against *β*-actin were purchased from Santa Cruz Biotechnology (Santa Cruz, CA, USA). Horseradish peroxidase-conjugated anti-mouse, -goat, and -rabbit IgG antibodies were purchased from BD Pharmingen Inc. (San Diego, CA, USA).

### 2.2. Cell Line, Cell Culture, and Drug Treatments

HA22T/VGH cell line was obtained from the Food Industry Research and Development Institute in Hsinchu, Taiwan (BCRC number: 60168) and was cultured in Dulbecco's modified Eagle's medium (DMEM) (Gibco BRL, Grand Island, NY, USA) containing 10% fetal bovine serum (FBS) (Hyclone, Auckland, NZ), 2 mM L-glutamine (Gibco BRL, Grand Island, NY, USA), 0.1 mM nonessential amino acids (Gibco BRL, Grand Island, NY, USA), 100 units/mL of penicillin, and 100 *μ*g/mL of streptomycin (Gibco BRL, Grand Island, NY, USA) at 37°C in a humidified chamber with 5% CO_2_. To investigate the effects of epirubicin and progesterone, various concentrations of epirubicin and progesterone were added to the culture medium for an indicated time period and then the cells were harvested and analyzed.

### 2.3. Cell Growth

After epirubicin and progesterone treatment, the cells were harvested and viable cells were counted using a dye exclusion technique as described previously [21]. Briefly, the cell suspension was centrifuged at 5,000 ×g; the supernatant was discarded, and the cell pellet was resuspended in serum-free medium. One volume of 0.4% Trypan blue (Gibco BRL, Grand Island, NY, USA) was added to one volume of cell suspension, and then cells were counted in a hemocytometer after incubation at room temperature for 3 min. All counts were done in triplicate.

### 2.4. TUNEL Assay

Terminal deoxynucleotidyl transferase-mediated dUTP nick-end labeling (TUNEL) assays were performed using an APO-BrdU TUNEL Assay Kit (Molecular Probes, Eugene, OR, USA) according to the manufacturer's directions as described previously [21]. Briefly, the cells were incubated for the indicated time before being trypsinized, washed with phosphate-buffered saline (PBS), and fixed in 2% paraformaldehyde (pH 7.4) for 15 min. The fixed cells were washed twice in PBS and stored at −20°C in 70% ethanol for 12–18 h prior to performing the TUNEL assay. After removing the 70% ethanol by centrifugation, the cells were washed twice in wash buffer and then incubated at 37°C for 60 min with DNA-labeling solution containing terminal deoxynucleotidyl transferase and BrdUTP. After washing twice with rinse buffer, the cells were resuspended for 30 min in the dark at room temperature in antibody solution containing Alexa Fluor 488-labeled anti-BrdU antibody. Flow cytometric analysis was subsequently performed using a Coulter Epics XL cytometer (Beckman Coulter, Miami, FL, USA) to quantify the fluorescence intensity for determination of apoptotic status. The data were analyzed using WINMDI software version 2.8 (Scripps Research Institute, La Jolla, CA, USA), with a minimum of 1 × 10^4^ cells per sample being evaluated in each case.

### 2.5. Caspase-3 Colorimetric Protease Assay

The activity of caspase-3 was detected using an ApoTarget caspase-3 colorimetric protease assay kit (Invitrogen Corp., Camarillo, CA, USA) according to the manufacturer's instructions as described previously [21]. Briefly, we induced apoptosis in cells by epirubicin and/or progesterone treatments while concurrently incubating a control culture without induction. We then counted cells as pellet 3–5×10^6^ cells per sample. The cells were resuspended in 50 *μ*L of chilled Cell Lysis Buffer, incubated on ice for 10 min, and then centrifuged for 1 min in a microcentrifuge (10000 ×g). Supernatant (cytosol extract) was transferred to a fresh tube and put on ice and protein assay reagents (Bio-Rad Laboratories, Hercules, CA, USA) were used. Each cytosol extract was diluted to a concentration of 50–200 *μ*g protein per 50 *μ*L Cell Lysis Buffer (1–4 mg/mL). A 50 *μ*L of 2 × reaction buffer (containing 10 mM DTT) was added to each sample followed by 5 *μ*L of the 4 mM DEVD-pNA substrate (200 *μ*M of final concentration). The samples were incubated in the dark at 37°C for 2 h. Samples were read in a microplate reader set at 405 nm. Fold increase in caspase-3 activity was determined compared to that in untreated controls.

### 2.6. Detection of Autophagy with Acridine Orange Staining

Formation of acidic vesicular organelles (AVOs), a morphological characteristic of autophagy, was quantified by acridine orange staining as described previously [22]. In brief, acridine orange (1 *μ*g/mL) was added 30 min prior to collection, and after being washed with PBS, cells were analyzed using the Coulter Epics XL cytometer (Beckman Coulter, Miami, FL, USA). Green (510–530 nm) and red (>650 nm) fluorescence emission from 1 × 10^4^ cells illuminated with blue (488 nm) excitation light was measured. The data were analyzed using WINMDI software version 2.8 (Scripps Research Institute, La Jolla, CA, USA), with a minimum of 1 × 10^4^ cells per sample being evaluated in each case.

### 2.7. Western Blotting

Sample preparation and Western blotting procedures were performed as described previously [21]. Briefly, cells were harvested and cytosolic extracts were prepared using lysis buffer (20 mM Tris-HCl (pH 7.2), 2 mM EGTA, 5 mM EDTA, 500 *μ*M sodium orthovanadate, 10 mM sodium fluoride, 1% Triton X-100, 0.1% SDS, and protease inhibitor cocktail). Protein concentrations were determined using protein assay reagents. Forty to sixty micrograms of protein lysate was analyzed by SDS-polyacrylamide gel electrophoresis. After transfer of the proteins from the gel to a nitrocellulose membrane (Amersham Pharmacia Biotech, Freiburg, Germany), the membranes were blocked for 1 h at room temperature in PBS with 0.05% Tween 20 (PBS-T) containing 5% nonfat dry milk, and then they were incubated with specific primary antibodies and horseradish peroxidase-conjugated secondary antibodies. The immunoreactive bands were visualized using an enhanced chemiluminescence kit (Perkin-Elmer Life Sciences, Boston, MA, USA).

### 2.8. Reverse Transcription-Polymerase Chain Reaction (RT-PCR)

Total RNA was extracted from cells with TRIzol reagent (Invitrogen Life Technologies, CA, USA) according to the manufacturer's instructions as described previously [23]. The complementary DNA (cDNA) was synthesized from random hexadeoxynucleotide primed reverse transcription from 2 *μ*g of total RNA using M-MLV reverse transcriptase (Promega Corporation, WI, USA) according to the manufacturer's directions. Polymerase chain reaction (PCR) was then performed using the Dream Taq DNA polymerase (Thermo scientific, MA, USA) on an Applied Biosystems Gene Amp9700 PCR system (Applied Biosystems, Foster, CA, USA). The thermocycling began with 94°C for 5 min followed by 30 cycles of 94°C for 1 min, 60°C for 1 min, and 72°C for 1 min, and then followed by 70°C for 10 min. PCR primers sequences were as follows: multidrug resistance-related protein 1 (MRP-1) forward, 5′-AGG TGGACCTGT TTC GTG AC-3′; reverse, 5′-ACCCTGTGATCCACCAGAAG-3′, and GAPDH forward, 5′-GAC ATC AAG AAG GTG GTG AAG CAG-3′; reverse, 5′-GCG TCA AAG GTG GAG GAG TGG-3′. The amplified PCR products were analyzed on 2% agarose gels and photographs were taken. The intensity of each band was calculated by densitometry analysis and the results were expressed as a percentage of the optical density of the corresponding GAPDH band.

### 2.9. Statistical Analysis

Comparisons among the groups of cells, one-way analysis of variance (ANOVA), and Fisher's least significant difference test were performed using the SPSS 17.0 statistical software (SPSS, Chicago, IL). All experiments were performed at least thrice. All data are expressed as the mean ± standard deviation (S.D.). Value differences were considered significant if *P* < 0.05.

## 3. Results and Discussions 

### 3.1. Inhibition of Cell Growth

**Figure 1:**
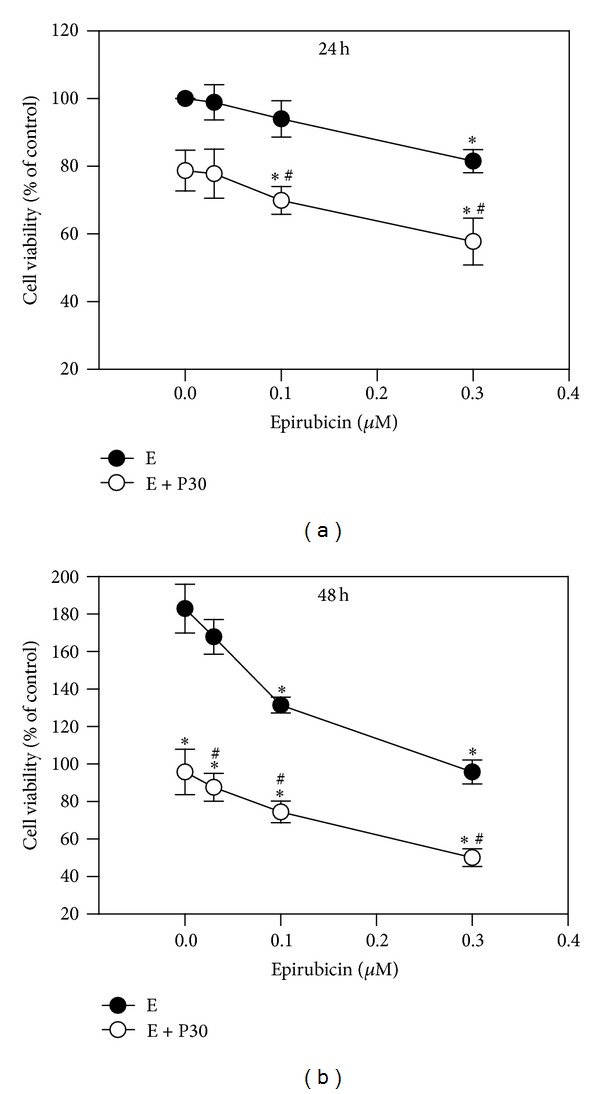
Effects of epirubicin on cell growth without or with progesterone addition. HA22T/VGH cells were treated with different concentrations of epirubicin without or with 30 *μ*M progesterone for 24 h (a) or 48 h (b). Results are expressed as the mean ± standard deviation (S.D.) for three separate experiments. E: epirubicin; P30: 30 *μ*M progesterone. *P < 0.05 compared to the corresponding untreated controls. ^#^ *P* < 0.05 compared to the corresponding epirubicin-treated group.

To gain initial insight into the effects of epirubicin alone or in combination with progesterone on cell growth of hepatoma cell line, HA22T/VGH cells were treated for 24 or 48 h without or with different doses of epirubicin in the absence or presence of 30 *μ*M progesterone. The IC_50_ of progesterone was 100 *μ*M and almost not cytotoxic to HA22T/VGH cells at concentrations <50 *μ*M (data not shown). Therefore, a concentration of 30 *μ*M progesterone was the dosage chosen for the cotreatment with epirubicin. During the 24 h incubation period, the untreated HA22T/VGH cells proliferated, while the growth of the cells treated with epirubicin ≥0.3 *μ*M was significantly inhibited. The addition of 30 *μ*M progesterone significantly augmented epirubicin's inhibition of growth at concentration of ≥0.1 *μ*M (Figure 1(a)). During 48 h incubation, epirubicin inhibited cell growth in a dose-dependent manner and the coaddition of progesterone augmented its effect (Figure 1(b)).

### 3.2. Induction of Apoptosis and Expressions of Apoptosis-Related Proteins

**Figure 2:**
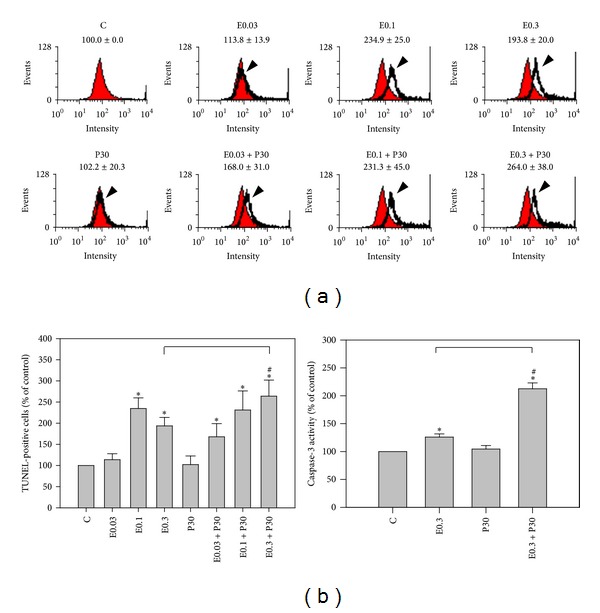
Apoptosis induction by epirubicin without or with progesterone addition. HA22T/VGH cells were treated with indicated concentrations of epirubicin without or with 30 *μ*M progesterone for 24 h and evaluated by (a) TUNEL staining or (b) caspase-3 activity. Results are expressed as the mean ± standard deviation (S.D.) for three separate experiments. C: untreated cells, E0.03: 0.03 *μ*M epirubicin, E0.1: 0.1 *μ*M epirubicin, E0.3: 0.3 *μ*M epirubicin, and P30: 30 *μ*M progesterone. *P < 0.05 compared to the untreated controls. ^#^ *P* < 0.05 compared to the corresponding epirubicin-treated group.

Apoptotic cells were measured by flow cytometric analysis after TUNEL staining (Figure 2(a)). After 24 h treatment with epirubicin, a significant increase in the percentage of TUNEL-positive apoptotic cells was seen as compared with controls at concentrations ≥0.1 *μ*M epirubicin. Although progesterone alone did not cause a significant change in the number of apoptotic cells, using it in combination with epirubicin at concentrations ≥0.3 *μ*M had a stronger effect. The fluorescence intensities of apoptotic cells of HA22T/VGH cells treated with 30 *μ*M progesterone, 0.3 *μ*M epirubicin, or combination therapy were 102.2 ± 20.3%, 193.8 ± 20.0%, and 264.0 ± 38.0%, respectively, compared with those of controls (Figure 2(a)).  Figure 2(b) shows that adding 30 *μ*M progesterone to 0.3 *μ*M epirubicin treatment produced more caspase-3 activity (212.5 ± 10.6%) than using 0.3 *μ*M epirubicin alone (126.0 ± 5.7%). These results showed epirubicin at concentration of 0.3 *μ*M activated caspase-3 and induced apoptosis, and combining the two drugs had a significant effect on apoptosis in HA22T/VGH cells.

**Figure 3:**
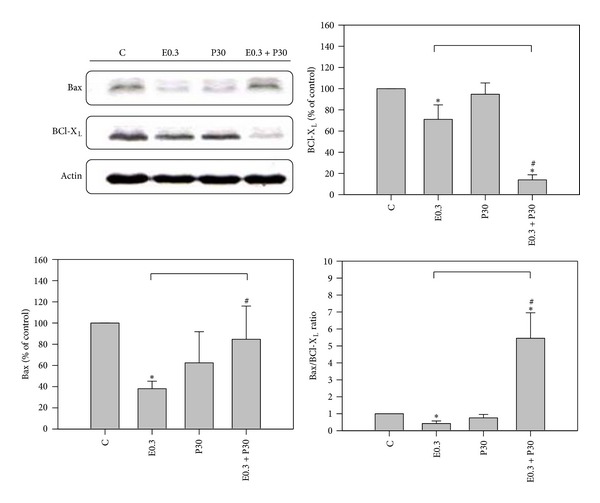
Western blotting shows Bax and Bcl-X_L_ expressions of HA22T/VGH cells after epirubicin and/or progesterone treatment for 24 h. Results are expressed as the mean ± standard deviation (S.D.) for three separate experiments. C: untreated cells, E0.3: 0.3 *μ*M epirubicin, and P30: 30 *μ*M progesterone. *P < 0.05 compared to the untreated controls. ^#^ *P* < 0.05 compared to epirubicin-treated group.

To determine whether the treatment-induced apoptosis was associated with altered expression of apoptosis-relating proteins, HA22T/VGH cells were treated for 24 h with 0.3 *μ*M epirubicin in the presence or absence of 30 *μ*M progesterone and analyzed by Western blotting.  Figure 3 shows that progesterone alone had no significant effect (94.8 ± 10.6% versus 100 ± 0.0%) on the antiapoptotic protein, Bcl-X_L_ levels, whereas epirubicin caused a decrease (71.1 ± 13.6% versus 100 ± 0.0%; *P* < 0.05), compared with those of controls. The combination of epirubicin and progesterone caused a marked decrease in expression of Bcl-X_L_ compared to 0.3 *μ*M epirubicin alone (13.9 ± 4.8% versus 71.1 ± 13.6; *P* < 0.05). Meanwhile, progesterone alone had no effect on the proapoptotic protein, Bax levels; epirubicin alone decreased Bax levels. The coaddition of progesterone and epirubicin significantly lessened the decrease of Bax levels compared to epirubicin alone (84.7 ± 31.3% versus 38.0 ± 7.1%; *P* < 0.05). Thus, the ratio of proapoptotic/antiapoptotic factor, Bax/Bcl-X_L_, was extremely enhanced by the combination therapy, which can partly explain why apoptosis was increased by the combination (Figure 3).

Activation of caspase-3 is an essential step in apoptosis. Our results demonstrated progesterone augmented caspase-3 activity of epirubicin-treated HA22T/VGH cells significantly. Epirubicin or doxorubicin can induce intracellular ROS [8, 9]. ROS production may interact with Fas-associated death domain (FADD) pathway and FADD sequence can result in activation of caspase-3 which has been reported in various cancer cell lines [24, 25]. This study also found that progesterone interfered with the expression of apoptosis-regulating proteins, upregulating Bax and downregulating Bcl-X_L_, in the epirubicin-treated HA22T/VGH cells. It is currently unknown whether progesterone initially triggers apoptosis upstream from caspase-3 or not. Bcl-X_L_ expression is important for the inhibition of apoptosis initiated by various cellular stresses in human HCC cells [26, 27]. We, therefore, propose that the Bcl-2 family may contribute to the improved efficacy of treating HA22T/VGH cells with a combination of epirubicin and progesterone. On the other hand, the expression of the progesterone receptor and its potential role in HA22T/VGH cells have not been reported till now; however, some studies have evaluated the role of the progesterone receptor-mediated apoptosis in other human hepatoma cells. Cheng et al. demonstrated that treatment with RU486, a progesterone receptor antagonist, inhibits the progesterone-mediated response to estradiol pretreatment in tumor necrosis factor-induced apoptotic Huh-7 cells [28]. On the contrary, Zhang and Chow reported that the progesterone receptor is not involved in the action of megestrol-induced apoptosis in HepG2 cells [29]. Thus, further studies on the potential role of the progesterone receptor in HA2T/VGH cells are necessary.

### 3.3. Autophagy Reduction by Combination

**Figure 4:**
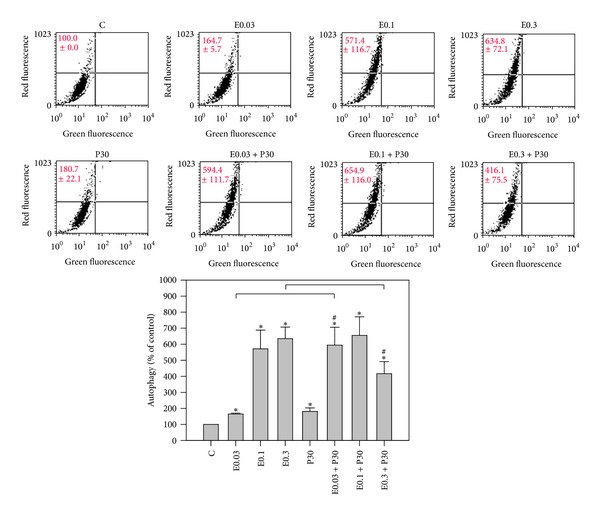
Epirubicin and/or progesterone effect on autophagy induction. HA22T/VGH cells were treated with epirubicin and/or progesterone for 24 h then stained with acridine orange and followed by flow cytometric analysis of autophagy. Results are expressed as the mean ± standard deviation (S.D.) for three separate experiments. C: untreated cells, E0.03: 0.03 *μ*M epirubicin, E0.1: 0.1 *μ*M epirubicin, E0.3: 0.3 *μ*M epirubicin, and P30: 30 *μ*M progesterone. *P < 0.05 compared to the untreated controls. ^#^ *P* < 0.05 compared to epirubicin-treated group.

**Figure 5:**
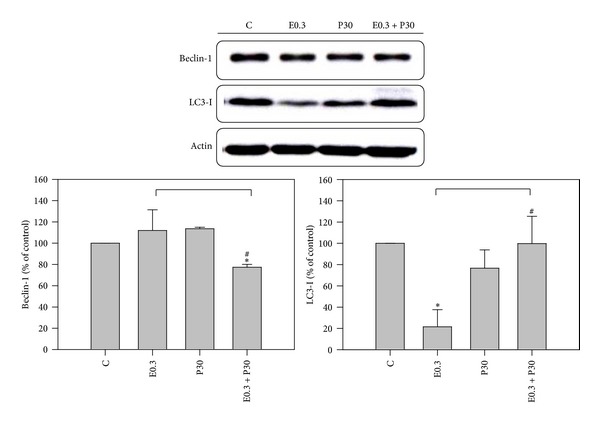
Western blotting shows Beclin-1 and LC3-I expressions in HA22T/VGH cells after epirubicin and/or progesterone treatment for 24 h. Results are expressed as the mean ± standard deviation (S.D.) for three separate experiments. C: untreated cells, E0.3: 0.3 *μ*M epirubicin, and P30: 30 *μ*M progesterone. *P < 0.05 compared to the untreated controls. ^#^ *P* < 0.05 compared to epirubicin-treated group.

It has been reported that autophagy can be induced during chemotherapy [30, 31]. To determine whether the treatments had an effect on autophagy induction, HA22T/VGH cells were treated for 24 h with 0.3 *μ*M epirubicin in the presence or absence of 30 *μ*M progesterone, then subjected to acridine orange staining, and analyzed by flow cytometry.  Figure 4 shows both epirubicin and progesterone increased autophagy compared to controls by AVOs analysis, though epirubicin was more effective than progesterone. Surprisingly, coaddition of progesterone significantly reduced the epirubicin-induced increase of autophagy. To further explore the expression of autophagy-related proteins, HA22T/VGH cells were treated for 24 h with 0.3 *μ*M epirubicin in the presence or absence of 30 *μ*M progesterone and then subjected to Western blotting. As shown in Figure 5, neither epirubicin nor progesterone had an effect on Beclin-1 levels, but the combination of the two significantly reduced Beclin-1 levels. The expressions of proteins of Beclin-1 of HA22T/VGH cells treated with 0.3 *μ*M epirubicin, 30 *μ*M progesterone, or combination therapy for 24 h were 119.9 ± 19.5%, 113.6 ± 1.4%, and 77.4 ± 2.6%, respectively, compared with those of controls. Figure 5 also shows progesterone had no effect on LC3-I levels, whereas epirubicin markedly reduced LC3-I levels, indicating that it may convert LC3-I to LC3-II. Interestingly, coaddition of progesterone to epirubicin treatment significantly reversed LC3-I levels. The expressions of proteins of LC3-I of HA22T/VGH cells treated with 0.3 *μ*M epirubicin, 30 *μ*M progesterone, or combination therapy for 24h were 21.5 ± 16.2%, 76.6 ± 17.3%, and 99.8 ± 25.6%, respectively, compared with those of controls. This is compatible with the results of AVOs formation shown in Figure 4.

Many studies have indicated that autophagy can serve as a survival mechanism for cancer cells and suggested that autophagy inhibitor might enhance the antitumor effects of chemotherapy or target therapy agents* in vivo* [30, 31]. In addition, Shen et al. reported inhibition of autophagy could enhance proapoptotic effects of ZD6474 in glioblastoma cells [31]. Greene et al. also reported inhibition of late-stage autophagy synergistically enhanced pyrrolo-1, 5-benzoxazepine-6-induced apoptotic cell death in human colon cancer cells [32]. In this study, progesterone was found to be able to reduce epirubicin-induced autophagy in HA22T/VGH cells. There are some interactions between autophagy and apoptosis mediated by Beclin-1 and Bcl-X_L_ proteins [32–34]. Beclin-1 is an autophagy-related protein, while Bcl-X_L_ is an anti-apoptosis-related protein. However, Bcl-X_L_ and Bcl-2 have been reported to be negative regulators of Beclin-1 [33–35]. Bcl-X_L_ can inhibit Beclin-1 activity by stabilizing Beclin-1 homodimerization [34]. Akar et al. reported that doxorubicin induced autophagy through the upregulation of Beclin-1, which was further enhanced by siRNA-mediated Bcl-2 silencing MCF-7 cells [36]. The expressions of Beclin-1 and AVOs were compatible in Figures 4 and 5, indicating that 30 *μ*M progesterone could decrease the expression of AVOs in HA22T/VGH cells treated with 0.3 *μ*M epirubicin and these effects may be induced by suppression of expression of Beclin-1. In contrast, the present study found that after progesterone was added, the expressions of Bcl-X_L_ and Beclin-1 both were reduced. Our results proved that progesterone in combination with epirubicin could increase the epirubicin-induced apoptosis and decrease epirubicin-induced autophagy in HA22T/VGH cells. Therefore, the decreased expression of Beclin-1 could not be explained by the interactions between Beclin-1 and Bcl-X_L_ described above.

Thus, Figure 4 displays progesterone increased autophagy compared to controls maybe due to the fact that 30 *μ*M progesterone does not augment the expression of Bcl-X_L_ shown in Figure 3. It has been demonstrated that the Toll-like receptor 4 (TLR4)—myeloid differentiation factor 88 (MyD88) pathway can mediate lipopolysaccharide (LPS)—induced autophagy by reducing the binding of Beclin-1 and Bcl-2 and thus triggers autophagy activation in human and murine macrophages [37, 38]. Su et al. reported progesterone inhibited TLR4-mediated innate immune response in murine macrophages [39]. Hepatocytes also express TLR4 receptors and are responsive to LPS [38, 40]. Our preliminary study also shows combination of progesterone and epirubicin can decrease epirubicin-induced expression of TLR4 and MyD88 and sequent production of interlukin-6 in HA22T/VGH cells (unpublished data). Thus, coaddition of progesterone significantly reduced the epirubicin-induced autophagy in HA22T/VGH cells which may be caused by inhibition of TLR4-MyD88 pathway by progesterone. However, further studies on the potential role of TLR4 and MyD88 pathway in HA22T/VGH cells are necessary.

The microtubule-associated protein 1-light chain-3 (LC3) is an ubiquitin-like molecule which is a mammalian homologue of the autophagy-related Atg8 encoded product in yeast [41]. During the fusion of autophagosomal membranes, cytosolic LC3 (LC3-I) is conjugated to phosphatidylethanolamine (PE) through two consecutive ubiquitylation-like reactions catalyzed by the E1-like enzyme (Atg7) and E2-like enzyme (Atg3) to form the LC3-phospholipid conjugate (LC3-II) [42, 43]. During the fusion of autophagosomes with lysosomes, intra-autophagosomal LC3-II is also degraded by lysosomal proteases [41, 43]. In this study, we found epirubicin alone enhanced the formation of AVOs but decreased the expression of LC3-I, whereas coaddition of progesterone decreased the formation of AVOs and reversed the expression of LC3-I. These results indicate that epirubicin may promote the turnover of LC3-I to LC3-II, a possibility that is compatible with epirubicin-induced formation of AVOs during the same incubation period (24 h). And addition of progesterone to epirubicin to treat HA22T/VGH cells significantly decreased epirubicin-induced autophagy.

### 3.4. Decrease of Multidrug Resistance-Related Protein 1 (MRP-1) mRNA Expression by Combination

**Figure 6:**
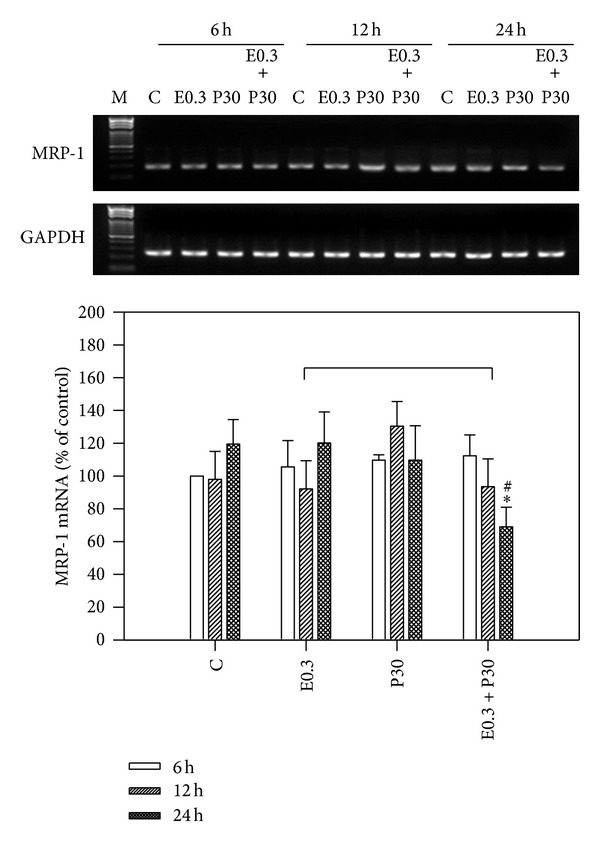
Epirubicin and/or progesterone effect on MRP-1 expression. HA22T/VGH cells were treated with epirubicin and/or progesterone for 6, 12, or 24 h, and then MRP-1 mRNA was analyzed by RT-PCR. The MRP-1 mRNA expression was normalized to GAPDH mRNA. The density of band was expressed as the relative density compared to that in untreated cells (control), taken as 100%. Results are expressed as the mean ± standard deviation (S.D.) for three separate experiments. C: untreated cells, E0.3: 0.3 *μ*M epirubicin, and P30: 30 *μ*M progesterone. *P < 0.05 compared to the untreated controls. ^#^ *P* < 0.05 compared to epirubicin-treated group.

We examined the MRP-1 mRNA expression in 0.3 *μ*M epirubicin and/or 30 *μ*M progesterone treated HA22T/VGH cells for 6, 12, or 24 h by RT-PCR analysis. As Figure 6 shows, there was no significant difference in MRP-1 mRNA expression by epirubicin and/or progesterone treatment for 6 h or 12 h, whereas cotreatment of epirubicin and progesterone produced lower MRP-1 mRNA expression after 24 h treatment, suggesting that the combination might lessen drug resistance in HA22T/VGH cells. The expressions of MRP-1 mRNA HA22T/VGH cells treated with 0.3 *μ*M epirubicin, 30 *μ*M progesterone, or combination therapy for 24 h were 120.1 ± 19.0%, 109.6 ± 21.0%, and 69.0 ± 12.0%, respectively, compared with those of controls. There are many studies indicating that chemotherapy can evoke drug resistance and that this resistance may be related to the expression of multidrug resistance-related protein gene, MRP-1 [44, 45]. Expression of multidrug resistance protein 1 (MRP-1) has been commonly observed in liver tissue and HCC cell lines treated with doxorubicin [46, 47]. It also has been reported that enhanced autophagy can induce drug-resistance in epirubicin-treated breast cancer cells [16] and increase of autophagy can induce production of MRP-1 [48]. This study found that the addition of progesterone to epirubicin-treated HA22T/VGH cells significantly decreased the expression of multidrug resistance-related protein 1 (MRP-1) gene, a decrease that might be related to the reduction of autophagy. Because the mechanisms underlying this possibility are not fully clarified in this study, more* in vitro* and* in vivo* studies are required.

## 4. Conclusions

Epirubicin is an anthracycline drug that can induce intracellular ROS [8, 9]. This study showed that epirubicin treatment inhibited the growth of HA22T/VGH cells in a dose-dependent manner. The addition of 30 *μ*M progesterone, which was inactive by itself, augmented epirubicin's inhibition of growth of cancer cells. Cotreatment with progesterone resulted in enhancement of the epirubicin-induced apoptosis, as evidenced by greater increase in caspase-3 activity and in the ratio of the apoptosis-regulating protein, Bax/Bcl-X_L_. The cotreatment also caused a decrease in autophagy, but decreased Beclin-1 and reversed LC3-I expressions. Furthermore, this combination reduced mRNA expression of the multidrug resistance-related protein 1 (MRP-1) gene.

However, clinical results of systemic single-epirubicin chemotherapy for palliative treatment of advanced HCC or prevention of postoperative recurrence in HCC have not been suggested [3, 6, 11, 12]. However, local embolization of unresectable or advanced HCC with epirubicin or doxorubicin through hepatic artery (TACE) has been suggested as a palliative treatment in west and east countries [3, 4]. Therefore, the present study shows the coadministration of epirubicin and progesterone might be a feasible and rational choice of therapy for clinical HCC treatment and this combination is worth further evaluation. However, more* in vitro* and* in vivo* studies are required.

## Abbreviations

HCC: Hepatocellular carcinoma
AVOs: Acidic vesicular organelles
FADD: Fas-associated death domain
MRP-1: Multidrug resistance-related protein 1
ROS: Reactive oxygen species
LC-3: Light chain-3
TLR-4: Toll-like receptor 4
MyD88: Myeloid differentiation factor 88.
